# Do patients adhere to over-the-counter artemisinin combination therapy for malaria? evidence from an intervention study in Uganda

**DOI:** 10.1186/1475-2875-11-83

**Published:** 2012-03-23

**Authors:** Jessica L Cohen, Elif Yavuz, Alexandra Morris, Jean Arkedis, Oliver Sabot

**Affiliations:** 1 Harvard School of Public Health, 677 Huntington Avenue, Boston, MA 02115, USA; 2 Brookings Institution, 1775 Massachusetts Avenue NW, Washington, DC 20036, USA; 3 Clinton Health Access Initiative, 383 Dorchester Avenue, Suite 400, Boston, MA 02127, USA; 4 Results for Development, 1100 15th Street NW, Suite 400, Washington, DC 20005, USA

## Abstract

**Background:**

Increasing affordability of artemisinin combination therapy (ACT) in the African retail sector could be critical to expanding access to effective malaria treatment, but must be balanced by efforts to protect the efficacy of these drugs. Previous research estimates ACT adherence rates among public sector patients, but adherence among retail sector purchasers could differ substantially. This study aimed to estimate adherence rates to subsidized, over-the-counter ACT in rural Uganda.

**Methods:**

An intervention study was conducted with four licensed drug shops in Eastern Uganda in December 2009. Artemether-lumefantrine (AL) was made available for sale at a 95% subsidy over-the counter. Customers completed a brief survey at the time of purchase and then were randomly assigned to one of three study arms: no follow-up, follow-up after two days or follow-up after three days. Surveyors recorded the number of pills remaining through blister pack observation or through self-report if the pack was unavailable. The purpose of the three-day follow-up arm was to capture non-adherence in the sense of an incomplete treatment course ("under-dosing"). The purpose of the two-day follow-up arm was to capture whether participants completed the full course too soon ("over-dosing").

**Results:**

Of the 106 patients in the two-day follow-up sample, 14 (13.2%) had finished the entire treatment course by the second day. Of the 152 patients in the three-day follow-up sample, 49 (32.2%) were definitely non-adherent, three (2%) were probably non-adherent and 100 (65.8%) were probably adherent. Among the 52 who were non-adherent, 31 (59.6%) had more than a full day of treatment remaining.

**Conclusions:**

Overall, adherence to subsidized ACT purchased over-the-counter was found to be moderate. Further, a non-trivial fraction of those who complete treatment are taking the full course too quickly. Strategies to increase adherence in the retail sector are needed in the context of increasing availability and affordability of ACT in this sector.

## Background

Artemisinin Combination Therapy (ACT) is the WHO recommended first-line therapy for *Plasmodium falciparum *malaria in nearly all malaria endemic countries and is the most effective treatment for the disease [1]. Several recent papers have stressed the urgency of developing strategies to stem parasite resistance to artemisinin, as resistant strains have already been detected [2-4]. The development of widespread resistance to previous generations of inexpensive, commonly purchased anti-malarials contributed to increases in malaria mortality among African children against a backdrop of overall declines in child mortality in this region [5,6].

Volumes of ACT purchased and distributed through private and retail sector channels are increasing substantially [7,8], though availability in retail outlets is still far from universal [9,10]. Since the majority of malaria treatment-seeking occurs in these sectors [11], and ACT is 5 to 24 times more expensive than alternative anti-malarials [9], price has been a major barrier to transitioning anti-malarial purchases from older, less effective medicines to ACT [12-15]. The Affordable Medicines Facility - malaria (AMFm) is a pilot initiative in seven African countries that aims to substantially reduce the price of ACT through subsidies [16]. Through this ACT price reduction, the AMFm also aims to encourage the purchase of ACT over artemisinin monotherapies (AMT), as AMT use can be a serious risk for parasite resistance to artemisinin-based drugs [17,18]. However, the AMFm's ultimate impact in protecting artemisinin will be inhibited if ACT is being used inappropriately [19].

Non-adherence to recommended treatment regimens, resulting in sub-therapeutic drug concentrations is a key driver of parasite resistance [17,20-22]. Non-completion of a full standard regimen also exposes patients to recurrent malaria infections and can increase related morbidity and mortality [23,24]. Previous studies have estimated a range of adherence rates to ACT obtained in the public sector, some finding nearly perfect adherence and others finding adherence rates as low as 39%, but with estimates varying importantly with the strictness of the definition, research methods and context [25-37]. It is questionable to what extent results from these studies can be generalized to patients purchasing ACT from private sector outlets such as licensed pharmacies, licensed and unlicensed drug shops, and other informal outlets like general stores and market stands. In most public sector studies, adherence is measured with home visits to patients who received ACT free-of-charge after a confirmed malaria diagnosis and often after receiving counseling, dosing instructions and side effects warnings from a trained provider who often supervised the first dose [25,28,29,32-34,37,38]. To the extent that diagnosis, instructions and supervision can improve adherence, retail sector adherence rates are likely to be lower [36]. Patient characteristics could differ importantly as well, e.g. if people are more likely to visit the public sector when the patient is young and the illness is severe [39,40]. Finally, retail sector customers may only be able to afford a sub-therapeutic dose of ACT and may choose to keep pills for the next time a household member is ill to defray future expense [41]. For all of these reasons, it may be inappropriate to forecast adherence rates to ACT provided through the retail sector based on existing public sector estimates.

As financing and other initiatives aiming to increase access to ACT reach the substantial share of malaria patients who seek treatment in the retail sector, it is increasingly crucial to have estimates of adherence rates among this population. Understanding ACT adherence in the retail sector is necessary for developing appropriate strategies to prevent the emergence of artemisinin resistance. This study estimates adherence rates and explores associated characteristics among patients purchasing over-the-counter ACT in Uganda. It is the first study to use follow-up methods to estimate adherence rates to subsidized, over-the-counter ACT.

## Methods

### Study area and context

The study was conducted in Soroti District, a rural area in Northeast Uganda, over three weeks between November and December of 2009. Soroti has a very high rate of malaria transmission, with more than 100 infective bites per person per year [42]. As common in the rest of Uganda [42,43], Soroti is a district in which patients frequently access medicines through the private sector, at formal or informal shops and pharmacies. Artemether-lumefantrine (AL) is the first-line anti-malarial for uncomplicated malaria in Uganda. At the time of the study, AL was approved for over-the-counter use, but less than 5% of drug stores carried ACT and the median retail price of ACT in drug stores was $8.76 [43]. The study took place roughly a year and a half before the introduction of the AMFm in Uganda. An ACT subsidy pilot program called the Consortium for ACT in the Private Sector Subsidy (CAPSS), run by Medicines for Malaria Venture (MMV) and the Ugandan Ministry of Health, took place in Uganda in 2008. Soroti was used as a control district for the evaluation of this pilot, but no interventions were conducted there.

### Study population

Brief surveys were conducted at 23 retail outlets ("drug shops") in Soroti, with information recorded on location, shop owner and attendant qualifications and years in business, customer traffic, days/hours open and most common anti-malarials sold. Four shops, all licensed and registered with Uganda's National Drug Authority, were selected from this sample to participate in the study. These shops were licensed "Class C" outlets, meaning that they can solely dispense over-the-counter medicines. Drug shops in Uganda are distinct from pharmacies, which can sell prescription medicines as well, but are typically located in urban areas. At the time of this study, ACT was in the process of being approved for over-the-counter dispensing in Uganda and we received special permission from the Ugandan Ministry of Health to allow our participating shops to dispense ACT. The shops that were included in the study were chosen because they were far apart from one another, staffed by well-qualified attendants, and were open a sufficient amount of time with enough customer traffic for us to obtain our patient sample in a three week time-frame. All four shops were managed by nursing assistants, were open all seven days a week (except one shop which was closed on Sunday) and were open from the early morning until mid-evening. The number of malaria patients visiting the shop per day ranged from 5 in one shop to 20 at the shop with the highest patient traffic. All shops cited Quinine as their most commonly sold anti-malarial. All four shops were located in the area of their village known as the trading center, which generally constitutes a grouping of a small number of retail outlets often including at least one drug shop. None of the shops were in a very remote location or in a busy town center.

Patients or their caregivers who purchased subsidized AL from the participating shops--either because they requested it or because the shop attendant recommended it--were recruited to enroll in the study immediately following purchase. Pregnant women and infants under four months of age were excluded from the study since AL is not approved for this patient population. Patients were recruited until enrollment reached 395, a sample size allowing us to detect a projected adherence rate in the three-day follow-up group of 70% with a 10% margin of error, a design effect of 2 and 20% loss to follow up.

### Intervention design and procedures

#### Enrollment

Shop attendants participated in a one-day training session led by a Uganda Ministry of Health-trained professional on storage, administration, and appropriate use of AL. With the information and materials obtained during the training, attendants were instructed to follow their normal dispensing practices. Shop attendants were trained to refer any patients who either did not recover after completion of anti-malarial treatment or who were exhibiting signs of severe malaria to the nearest public health facility.

Following the training, the study team provided the selected drug shops with a controlled supply of AL, brand name Coartem^® ^manufactured by Novartis, for sale in the shops. The Coartem treatment package was the standard packaging commonly available in the private sector, which is a simple orange and white box with black and red text on it. Unlike some packaging provided in the public sector, this packaging included no drawings or visual cues on appropriate dosing other than a paper insert with written English instructions printed in black and white. The price of Coartem to consumers was fixed at 200, 400, 600 or 800 Ugandan Shillings ($0.10 - $0.40 at 2009 exchange rates) depending on the age/weight band. Shops were permitted to keep 50 Ugandan Shillings on every sale. Prices were equivalent to those in the CAPSS ACT subsidy pilot programme [44] and roughly equal to the final retail prices targeted under the AMFm [45]. A full course of AL consists of six doses, with 1-4 tablets per dose, depending on bodyweight/age, administered over the course of three days [46].

All eligible customers who purchased AL and gave consent to be surveyed at the shop were enrolled. The study team conducted a brief survey at the shop to collect symptom and background information (including the village and location of the household) and to record observations regarding the interaction between the shop attendant and the customer. If the customer was purchasing AL for another family member or neighbour, the household information for both the customer and the patient was obtained. Neither customers nor patients were informed of the intent to follow-up or were asked to retain their blister pack. All questionnaires and informed consent documents were translated into Ateso, the predominant language in Soroti. Interviews and informed consent processes were conducted in either English or Ateso, according to the language preferences of the participant.

#### Randomization assignment

A pseudo-random number generator in STATA Version 11 (STATA Corporation, College Station, Texas) was used to create a list that assigned a follow-up assignment to each patient, based on the order in which they were enrolled. The project manager had one list numbered 1 to 100 for each shop, with a pre-filled follow-up assignment for each number. Surveyors enrolling patients at the shop (and the patients themselves) were blinded to the eventual follow-up assignment. Participants were randomly assigned to either no follow-up, follow-up at day two or follow-up at day three. The aim of the three-day follow-up was to assess whether patients delayed or discontinued treatment. The aim of the two-day follow-up was to assess whether patients finished their entire treatment course too early. The aim of the no follow-up arm was to introduce additional uncertainty about the intent to follow up. The window of time following enrollment for the two-day follow-up arm was between 41 and 55 hours, while for the three-day follow-up arm it was between 72 and 96 hours.

#### Follow-up visits

At the household follow-up visit, patients or their caregivers were again asked for consent to be surveyed. Follow-up questionnaires included sections on demographic and health background information and household assets in order to characterize socioeconomic status. Additional sections focused on the patient's medicine-taking experience and behaviour, during which time the surveyor asked to see the blister pack and recorded the number of tablets remaining in the pack. If the pack was unavailable or the participant declined to show the pack, the surveyor asked the participant how many pills were remaining. Finally, surveyors reviewed appropriate adherence practices with the participant. If participants were still feeling ill at the time of the household visit, enumerators were instructed to recommend to patients that they visit the nearest public health facility as soon as possible.

#### Definition of adherence

Adherence is defined with regard to completion of the entire age-appropriate treatment course at the three-day follow-up visit, based on blister pack observation or, in its absence, self-report. A patient was considered "probably adherent" if the blister pack was available for inspection and there were no pills left or if no blister pack was available but the patient reported that all the pills were taken. A patient was considered "definitely non-adherent" if the blister pack was available for inspection with pills remaining in the pack and "probably non-adherent" if no blister pack was available for inspection but the patient reported that the treatment course was not completed.

#### Ethical considerations

The study gained approval from the Uganda National Council for Science and Technology (UNCST), Uganda's Office of the President, and the Harvard School of Public Health Institutional Review Board.

#### Analysis

Survey data were double entered in CSPro Version 3.1 (U.S. Census Bureau. Washington D.C., USA) and crosschecked for discrepancies until the error rate calculated for all sections was below 0.5%. The investigators cleaned and analysed data using STATA Version 11 software (STATA Corp., College Station, Texas). Analysis of variables associated with adherence is conducted in two ways. First, running probit regressions of the dichotomous variable "probably adherent" (as defined above) on each of the variables listed in Tables 1 and 2. For this analysis, coefficient estimates are reported as marginal effects of the independent variables on the probability of adherence. The second type of analysis is a series of ordinary least squares regressions with a variable measuring the number of doses remaining at follow-up (including zero if the patient adhered) as the dependent variable and again with each of the variables listed in Tables 1 and 2 as independent variables. All regressions control for the shop at which the transaction took place, since the randomization was stratified by shop [47].

**Table 1:** Characteristics of Sample and of Drug Purchase Transaction

	Two-Day Follow-Up, Mean (SD)	Three-Day Follow-Up, Mean (SD)
Observations	106	152
Customer Age (years)	33.4 (11)	33.6 (13)
Customer Sex (Female)	59.4% (49)	57.6%(50)
Customer and Patient Are Same	28.3% (45)	26.7% (44)
***Characteristics of Patient/Caregiver***		
Patient Age (years)	16.4 (19)	17.3 (22)
Patient is 3 years or Below	32.1% (47)	40.8%(49)
Patient is 4-7 Years	26.4%(44)	15.1% (36)
Patient is 8-12 Years	4.7% (21)	7.2%(26)
Patient is 13 Years or Older	36.8% (48)	36.8%(48)
Patient Sex (Female)	59.4% (49)	53.3% (50)
Number of Dependents	5 (3)	5 (3)
Married	84.9% (36)	84.2% (37)
Reads English	39.6% (49)	41.7% (49)
Years of Education	5.5 (4)	5.4 (4)
Some Secondary Education or Beyond	16% (37)	18.4% (39)
Subsistence Farmer	70.8% (46)	77% (42)
***Characteristics of Patient/Caregiver's House and Assets***		
Distance from Drug Shop (Km; assessed by respondent)	2.1 (2)	2.6 (8)
Land (Acres)	3.6 (3)	3.7 (3)
No Toilet	34.9% (48)	33.6% (47)
Iron Roof	17% (38)	22.4% (42)
Number of Cattle Owned	2.6 (4)	2.9 (6)
Number of Chickens Owned	7.3 (9)	7.1 (9)
***Health Behavior***		
Number of Mosquito Nets Hanging	2.5 (1)	2.4 (1)
First Child Born in Facility (among those with children)	40.4% (49)	36.7% (48)
Treat/Purify Water	9.4% (29)	13.3% (34)
***Characteristics of This Illness Episode***		
Visited a Health Facility Before Shop	23.6% (43)	24% (43)
Days Waited Before Seeking Any Treatment	2.8 (4)	2.9 (4)
Medicines Taken Before Visiting Shop	50.9% (50)	52.3% (50)
Patient Reports Having Fever	58.5% (50)	55.0 (50)
***Familiarity with and Perceptions of Coartem***		
Stated Reason for Purchasing Coartem at Shop: "Believe it is Most Effective"	51.9% (50)	51.3% (50)
Stated Reason for Purchasing Coartem at Shop: "Taken it Before and Liked It"	25.5% (44)	27.0% (45)
Stated Reason for Purchasing Coartem at Shop: "Shop Attendant Recommended"	22.6% (42)	29.6% (46)
If Money Were No Concern, Patient would Choose Coartem	90.2% (30)	82.8% (38)
If Would Choose Coartem, Why: "Believe it is Most Effective"	75.0% (44)	80.8% (40)
If Would Choose Coartem, Why: "Taken it Before and Liked It"	30.4% (46)	30.0% (46)
If Would Choose Coartem, Why: "Shop Attendant Recommended"	10.9% (31)	8.3% (28)
***Illness Resolution***		
Still Experiencing Fever	21.7% (41)	21.5% (41)
Condition Has Improved	93.9% (24)	90.5% (29)
Patient is Able to Work/Play	93.3% (25)	91.8% (27)
***Perceptions of Malaria Incidence: "Chances of Getting Malaria in the Next Month (Out of 10)"***		
Adults	5.1 (3)	4.8 (3)
Child Age 6-15	6.5 (3)	6.6 (3)
Child Under 2 Years	7.3 (3)	7.4 (3)

**Table 2:** Characteristics of Drug Shop Transaction

	Two-Day Follow-Up, % (SD)	Three-Day Follow-Up, % (SD)
*Observed*		
Patient Got Correct Dose for Age	96.2% (19)	94.7% (22)
Patient Paid Recommended Price	100% (0)	99.3% (8)
Shop Attendant Gave Complete Dosing Instructions	78.5% (41)	82.0% (39)
Shop Attendant Gave Instructions in Case of Vomiting	16.3% (37)	18.8% (39)
Shop Attendant Gave Instruction in Case the Patient Gets Worse	3.8% (19)	4.0% (20)
Shop Attendant Gave Instruction in Case the Patient Does not Improve	4.8% (21)	5.4% (23)
*Reported by Patient/Caregiver*		
Shop Attendant Gave Verbal Instructions	90.5% (29)	82.1% (38)
Verbal Instructions Were Clear (If given)	99.0% (10)	98.4% (13)
Shop Attendant Gave Written Instructions	83.8% (37)	88.4% (32)
Written Instructions Were Clear (If given)	47.7% (50)	50.8% (50)

The shop attendant was considered to have given complete instructions if instructions were given on how many tablets should be taken with each dose, the number of times per day each dose should be taken and the number of days the doses should be taken. Patients/caregivers were only asked whether or not they received any verbal or written instructions but were not asked to specify which instructions they were given.

Variables potentially associated with adherence are presented in Tables 1 and 2. These include (Table 1) socio-economic status measures (age, gender, marital status, occupation and assets), education measures (years of education, level of education and literacy), health behaviour measures (bed net usage, water purification and health system usage), treatment-seeking for this illness episode (how long the patient waited before seeking any care and whether or not a health facility was visited or medicines were taken prior to visiting the drug shop), illness resolution measures (whether the condition has improved and the patient can work or play at follow-up and whether the patient is still experiencing fever), and perceptions of malaria incidence. Perceptions of malaria incidence were elicited since this could influence the incentive to save pills for future malaria episodes. Respondents were asked, for different age groups, how many babies/children/adults out of 10 they would expect to get malaria in the next month. Characteristics of the drug shop transaction (such as what instructions were given and whether they were clear) were also recorded based on both direct observation at the drug shop and self-report by the patient at the follow-up visit.

## Results

### Description of population and drug shop transaction

Of the 395 enrolled customers, 39 (9.9%) were assigned to no follow-up, 159 (40.3%) were assigned to two-day follow-up and 197 (49.9%) were assigned to three-day follow-up. Loss to follow-up was balanced across groups at 27 (17%) and 32 (16.2%) in the two and three-day follow-up groups, respectively. Since the timing of the survey and blister pack observation is critical for accurate adherence data, those found beyond the appropriate time were excluded from the analysis. This includes 26 (16.4%) patients in the two-day follow-up group and 13 (6.6%) in the three-day follow-up group, leaving a sample of 106 patients in the two-day group and 152 in the three-day group. Among those found on time 91.5% (n = 97) and 73.7% (n = 112) showed their blister pack for inspection in the two-day and three-day groups, respectively. The most common reasons for not showing the blister pack in the three-day group were that it was thrown away (58.7%) or lost (30.4%).

The randomization successfully achieved balance across observable characteristics (Table 1)--the two follow-up groups only differed significantly (p = .025) in the share of patients ages four to seven years. The majority of patients in the sample were from age groups prescribed the highest (age 13 and over; ~36%) and lowest (three months through three years; ~32-40%) doses of AL. The patient and customer were different in over 70% of cases, but the great majority of these cases were adult customers buying medication for a child who was the patient. When the patient and customer were not the same, the average age of the patient was 8 and 84% of the patients were 10 or younger. Since the AL packaging was written in English, it is important to note that only roughly 40% of patients or their caregivers could read English. Patients lived on average just over 2 kilometers from the drug shop and were for the most part subsistence farmers with low levels of education. Only about one-quarter of patients visited a health facility prior to arriving at the shop but more than half had taken some other medicines before this shop visit.

Although this study was conducted prior to the AMFm, familiarity with Coartem was high in this population, possibly because of experience with the drug through the public sector. When asked at the follow-up visit why the patient or caregiver purchased Coartem half of respondents answered that they believed it was the most effective anti-malarial and a quarter responded that they had taken it before and liked it. The majority of patients/caregivers, when asked which anti-malarial they prefer if money were no concern stated Coartem (90% and 83% in the two- and three-day follow-up groups, respectively) and again the most commonly stated reasons for this preference were because it was believed to be most effective or because they had taken it before and liked it. Nearly all patients said their condition had improved and they could work or play at follow-up, but just over 20% said they were still experiencing some fever. Respondents' expectations of the likelihood of malaria in the next month were very high--roughly 50% chance of infection in the next month for adults and nearly 75% chance for babies.

Nearly all patients received the correct dose for their age group and paid the correct price (Table 2). Those who received the incorrect dose were either on or very close to the boundary of one of the age bands (Additional file 1 Table S1). Adherence among these patients is thus assessed on the basis of the number of pills taken from the pack size they actually received in the results below. Based on shop observation, attendants provided patients with complete instructions on dosing, including the number of pills per dose, the number of doses per day and the number of days for treatment, approximately 80% of the time. However, instructions on what to do in case of vomiting or if the patient got worse or did not improve were given a minority of the time. Based on patient or caregiver report, just over 80% of respondents in the three-day follow-up group claimed that the shop attendant gave them at least some verbal dosing instructions and nearly everyone who received verbal instructions said that these instructions were clear. 88% of patients in the three-day follow-up group claimed that they were provided with written instructions, but only half of respondents said that these written instructions were clear. Patient/caregiver reports about receipt and clarity of instructions were similar in the two-day follow-up group.

### Adherence

Just over 13% (14/106) of patients had finished all of their medicine at the time of the home visit in the two-day follow-up group. The mean number of doses left on day two was 1.67 (95% CI 0, 5).

Out of 152 patients in the three-day follow-up group, 100 (65.8%) were "probably adherent", whereas 49 (32.2%) were "definitely non-adherent" and 3 (2.0%) were "probably non-adherent." Among the non-adherent group (n = 52), the average number of doses left was 2.27 (95% CI .33, 6). 31 (59.6%) patients in the non-adherent group had more than a full day of treatment remaining (Table 3).

**Table 3:** Adherence Behaviour (Three-Day Follow-Up Group)

	**All (n = 152)**,
	Mean (SD)
Probably Adherent	65.8% (48)
Definitely Non-Adherent	32.2% (47)
Probably Non-Adherent	2.0% (14)
	Non-Adherent Group (n = 52), Mean (SD)
Mean Number of Doses Remaining	2.27 (1.50)
More than One Full Day of Treatment Remaining	59.6% (50)

A "dose" refers to a half-day of treatment for the patient's age group. A full treatment course consists of 6 doses for all ages

### Variables associated with adherence

No significant relationship was found between the age or gender of the patient and whether or not treatment was completed (Table 4), although female patients had .47 fewer doses left than male patients (p = .036; 95% CI [-.917, -.03]). None of the socioeconomic status variables had any significant relationship to treatment completion or doses left other than the variables measuring education. Patients or their caregivers who attained some secondary school education or beyond were 22% more likely to complete the treatment (p = .024; 95% CI [.063, .382]). Those able to read English had .47 fewer doses left (p = .049; 95% CI [-.93, -.003]). None of the variables related to health behaviour, including the number of bed nets hanging in the household, were significantly associated with adherence.

**Table 4:** Associations with Adherence Behaviour (Three-Day Follow-Up Group)

	Dependent Variable:					
	A. Probably Adherent (Marginal Probit)			B. Number of Pills Remaining (Ordinary Least Squares)		
	Coefficient on Independent Variable	Standard Error	P-Value	Coefficient on Independent Variable	Standard Error	P-Value
Customer Age (years)	0.001	(0.003)	[0.749]	-0.006	(0.009)	[0.540]
Customer Sex (Female)	0.006	(0.080)	[0.942]	0.102	(0.232)	[0.663]
Customer and Patient Are Same	0.044	(0.087)	[0.611]	-0.299	(0.258)	[0.249]
***Characteristics of Patient/Caregiver***						
Patient Age (years)	-0.001	(0.002)	[0.599]	-0.002	(0.005)	[0.646]
Patient is 3 years or Below	0.038	(0.080)	[0.638]	0.104	(0.233)	[0.658]
Patient is 4-7 Years	-0.110	(0.113)	[0.329]	0.414	(0.316)	[0.192]
Patient is 8-12 Years	0.162	(0.150)	[0.279]	-0.285	(0.437)	[0.515]
Patient is 13 Years or Older	-0.026	(0.081)	[0.747]	-0.252	(0.235)	[0.287]
Patient Sex (Female)	0.074	(0.079)	[0.344]	-0.474	(0.225)	[0.036]
Number of Dependents	0.004	(0.012)	[0.735]	-0.026	(0.036)	[0.472]
Married	0.108	(0.109)	[0.325]	0.094	(0.311)	[0.762]
Reads English	0.067	(0.083)	[0.417]	-0.467	(0.235)	[0.049]
Years of Education	0.013	(0.010)	[0.213]	-0.045	(0.028)	[0.106]
Some Secondary Education or Beyond	0.223	(0.099)	[0.024]	-0.504	(0.289)	[0.083]
Subsistence Farmer	-0.110	(0.094)	[0.241]	0.069	(0.276)	[0.804]
***Characteristics of Patient/Caregiver's House and Assets***						
Distance from Drug Shop (Km; assessed by respondent)	0.005	(0.008)	[0.556]	-0.011	(0.016)	[0.504]
Land (Acres)	-0.023	(0.015)	[0.120]	0.058	(0.040)	[0.155]
No Toilet	-0.013	(0.085)	[0.878]	-0.009	(0.252)	[0.972]
Iron Roof	0.061	(0.101)	[0.544]	0.123	(0.289)	[0.671]
Number of Cattle Owned	0.005	(0.008)	[0.572]	-0.011	(0.020)	[0.579]
Number of Chickens Owned	0.007	(0.006)	[0.197]	-0.018	(0.013)	[0.166]
***Health Behavior***						
Number of Mosquito Nets Hanging	0.053	(0.034)	[0.116]	-0.120	(0.088)	[0.176]
First Child Born in Facility (among those with children)	0.013	(0.083)	[0.872]	0.012	(0.247)	[0.960]
Treat/Purify Water	-0.109	(0.117)	[0.352]	0.370	(0.335)	[0.272]
***Characteristics of This Illness Episode***						
Visited a Health Facility Before Shop	-0.003	(0.092)	[0.974]	-0.020	(0.269)	[0.941]
Days Waited Before Seeking Any Treatment	-0.020	(0.011)	[0.080]	0.030	(0.029)	[0.299]
Medicines Taken Before Visiting Shop	-0.096	(0.078)	[0.219]	0.121	(0.229)	[0.597]
Patient Reports Having Fever	-0.059	(0.079)	[0.453]	0.143	(0.230)	[0.537]
***Familiarity with and Perceptions of Coartem***						
*Stated Reason for Purchasing Coartem at Shop:*						
"Believe it is Most Effective"	0.065	(0.079)	[0.407]	-0.375	(0.227)	[0.101]
"Taken it Before and Liked It"	0.120	(0.089)	[0.178]	-0.098	(0.265)	[0.711]
"Shop Attendant Recommended"	-0.043	(0.086)	[0.618]	-0.027	(0.253)	[0.916]
If Money Were No Concern, Patient would Choose Coartem	0.155	(0.106)	[0.143]	-0.257	(0.281)	[0.362]
*If Would Choose Coartem, Why:*						
"Believe it is Most Effective"	-0.047	(0.108)	[0.660]	0.133	(0.291)	[0.648]
"Taken it Before and Liked It"	0.098	(0.093)	[0.293]	-0.074	(0.265)	[0.781]
"Shop Attendant Recommended"	-0.045	(0.149)	[0.761]	0.265	(0.413)	[0.522]
***Illness Resolution***						
Still Experiencing Fever	-0.140	(0.096)	[0.147]	0.449	(0.247)	[0.070]
Condition Has Improved	0.257	(0.142)	[0.070]	-0.634	(0.356)	[0.077]
Patient is Able to Work/Play	0.093	(0.148)	[0.529]	-0.424	(0.381)	[0.268]
***Perceptions of Malaria Incidence: "Chances of Getting Malaria in the Next Month (Out of 10)"***						
Adults	-0.026	(0.016)	[0.117]	0.053	(0.046)	[0.245]
Child Age 6-15	-0.030	(0.015)	[0.049]	0.088	(0.043)	[0.042]
Child Under 2 Years	-0.020	(0.014)	[0.140]	0.037	(0.038)	[0.326]
***Instructions Given by Shop Attendant (Observed)***						
Complete Dosing Instructions	-0.049	(0.113)	[0.662]	0.137	(0.346)	[0.692]
Instructions in Case of Vomiting	-0.052	(0.103)	[0.614]	-0.254	(0.299)	[0.396]
Instruction in Case the Patient Gets Worse	-0.374	(0.218)	[0.086]	0.424	(0.583)	[0.467]
Instruction in Case the Patient Does not Improve	-0.166	(0.179)	[0.354]	0.222	(0.508)	[0.663]
***Instructions Given (Reported by Patient/Caregiver)***						
Shop Attendant Gave Verbal Instructions	0.281	(0.106)	[0.008]	-0.807	(0.282)	[0.005]
Shop Attendant Gave Written Instructions	0.151	(0.129)	[0.242]	-0.455	(0.363)	[0.212]
Written Instructions Were Clear (If given)	0.165	(0.082)	[0.045]	-0.635	(0.214)	[0.004]

Estimates in Panel A are from probit regressions of a binary variable for whether or not treatment was completed on the independent variable indicated by row. Coefficient estimates from these regressions are reported as marginal effects. Estimates in Panel B are from linear ordinary least squares regressions of a variable indicating the number of pills remaining (including zero if the patient finished treatment) on the independent variable indicated by row. The variable indicating clarity of verbal instructions (Table 2) is not included here because only 2 people in the three-day follow-up group claimed that instructions were unclear

While the coefficient estimates are not quite significant at conventional levels, the results are suggestive of a relationship between the condition of the patient at the time of follow-up and adherence. Those whose condition had improved were 26% more likely to have completed treatment (p = .070; 95% CI [-.026, .54]) and had .63 fewer doses remaining (p = .077; 95% CI [-1.33, .069]) than those whose condition had not improved. Those still experiencing fever had .45 more doses left (p = .07; 95% CI [-.038, .936]) than those whose fever had resolved. Higher expected likelihood of malaria in the next 30 days is significantly associated with lower levels of treatment completion and more doses left for children ages 6-15 (p = .049 and 95% CI [-.059, -.000] for treatment completion; p = .042 and 95% CI [.003, .172] for doses left).

The data are mixed on the relationship between shop attendant instructions and adherence behavior. There is no significant relationship between treatment completion or the number of pills left and any of the variables measuring instructions given at the shop based on observation. However, we also asked patients (or their caregivers) at the time of follow-up whether or not they were provided with instructions and whether or not those instructions were clear. Based on these self-reported measures, having received instructions is associated with a 28% increase in treatment completion (p = .008; 95% CI [.071, .49]) and .81 fewer doses remaining (p = .005; 95% CI [-1.36, -.25]). Further, among patients who received written instructions, those reporting that the instructions were clear were 16.5% more likely to complete treatment (p = .045; 95% CI [.006, .324]) and had .64 doses fewer remaining (p = .004; 95% CI [-1.06, -.212]) than those reporting that the instructions were unclear.

## Discussion

A substantial amount of research has been conducted on adherence to ACT obtained through the public sector with generally encouraging results [24,26,28,29,33,34] overall. Several important recent public sector studies, however, find adherence results close to or lower than the levels we report here [25,27,35,37,38]. It is hard to infer adherence rates among retail sector patients from these studies, however, since the types of patients, illnesses and services provided in the retail sector could differ substantially. Results from this study suggest that adherence rates to ACT sold through the retail sector are moderate, with 65.8% of patients "probably adherent." While patients' commonly stated that their intention was to finish the remaining pills, 59.6% of those who had any pills left had more than one full day of treatment remaining, suggesting that the lack of adherence was unlikely due to premature visits to patients who would have completed the final dose soon thereafter. A stricter definition that included the timing of dosing would have yielded lower adherence rates.

Follow-up home visits from the two-day follow-up group revealed that 13% of patients had already finished their medicine. This is a non-trivial fraction of patients, but it is low enough to infer that, for the most part, people are aware that the treatment should not be completed by day two. Combined with the fact that we observed shop attendants giving dosing instructions in the majority of cases, this suggests that non-adherence is less likely due to a general non-comprehension of dosing than to a non-comprehension of the importance of finishing the treatment course specifically, a message that should perhaps be emphasized in drug shop training or other interventions to increase adherence. Adherence rates are found to be higher among patients whose condition has improved and who are no longer experiencing fever, possibly suggesting that people stop treatment when they feel it is not helping. It is also possible that this association between adherence and illness resolution is simply due to the fact that finishing the entire course leads people to feel better. Shop attendants in this study very rarely provided instructions in case the illness got worse or did not resolve and it is possible that this lack of detailed instruction contributed to the decision to discontinue treatment. Further, it seems likely that verbal and written instructions in the retail sector need to be made more understandable since: 1) non-adherence is significantly more likely among those reporting that they did not receive clear instructions, 2) the packaging insert was in English, while the majority of patients could not read English, and 3) education was significantly correlated with adherence. Patients who anticipate frequent malaria infections were found to be less likely to adhere, suggestive that the adherence decision could be related to the desire to keep pills for the next malaria episode.

The setting and characteristics of the study could affect the generalizability of these results to retail sector ACT purchasers in other countries and contexts. ACT was very heavily subsidized. To the extent that price influences adherence (e.g. because people can only afford partial doses or save some pills to defray the high future expense), it may not be appropriate to infer adherence rates from this study to areas in which ACT is unsubsidized or is subsidized but has a substantially higher retail price. While we show in Table 1 that familiarity with and preference for ACT was high in our study population, it could also be the case that as the AMFm has rolled out in Uganda, familiarity with ACT dosing has increased, perhaps increasing adherence rates. The fact that shop attendants in our study were chosen based on their qualifications, were given training on ACT dispensing and were under observation from our study staff most likely led to the high rates of written and verbal instructions provided and to the nearly perfect administration of dosing by age group. Other research finds that retail shops often provide sub-therapeutic doses of ACT, even when it is pre-packaged [48]. This suggests that the estimates of adherence in the retail sector found in this study may be upper bounds. Similarly, while efforts were made to limit patient awareness of the intention to follow-up with them, it is possible that adherence rates were higher than would truly exist among retail patients who were not part of a research study.

The sample sizes used for the analysis were smaller than ideal (and thus confidence intervals were wide) because of the loss to follow-up and the fact that people were found beyond the appropriate time frame. This attrition was largely due to the fact that patients were not made aware of the intention to come to their household for a follow-up visit and were not asked for directions to the household, a trade-off made to reduce the degree to which patients adhered to treatment because they knew they were under observation. Finally, blister packs were observed for roughly three-quarters of the sample. Those who did not show blister packs could have thrown them away because they had finished treatment or could have been reluctant to show us the packs because pills were left over. If the latter is the case, our estimates of adherence are too high.

The retail sector is the most common source of anti-malarials in general, and increasingly a common source for ACT. Nearly 150 million subsidized courses of ACT have been purchased through the AMFm for retail sector distribution, an unprecedented scale and speed of a public intervention to finance distribution of a drug in the private sector in the developing world. At the end of 2012, informed by the results of an independent evaluation, the Global Fund Board will make a critical policy decision on whether to continue with this initiative or potentially expand it. The first phase of the AMFm has included some interventions which could increase ACT adherence, such as additional ACT packaging requirements and training of private retail staff. ACT manufacturers participating in the AMFm were required to create separate packaging by age/weight band, to clearly mark the individual dose sub-units and to provide symbolic representations of key instructions. As our adherence estimates pertain to a sample of patients who received ACT from trained shop attendants who administered the correct (age-appropriate) dose in nearly every case, these findings suggest that additional measures may be needed to achieve high adherence rates. Any such measures will need to be highly cost-effective and operationally simple given the constraints on global health resources and the scale of private sector ACT distribution. Options could include enhanced simple information or instructions on ACT packaging and mobile phone based reminders to patients, both of which are being examined through research being implemented in connection with the AMFm [49]. Results from this study suggest that adherence may be an important factor in considering both the impact and future design of the AMFm and other initiatives striving to increase access to ACT while preserving its effectiveness.

## Competing interests

OS and AM are members of, and JC receives research support from, the Clinton Health Access Initiative, which is actively supporting efforts to expand funding for and implementation of ACT treatment in the private sector.

## Authors' contributions

JC, JA and OS conceptualized and designed the study. JC was Principal Investigator and AM and JA managed study implementation. JC, EY and AM conducted data analysis. All authors contributed to the manuscript. All authors read and approved the final manuscript.

## Supplementary Material

Additional file 1 **Table S1**. Dosage Given by Patient Age. Click here for file
