# Supplementary material for: Do patients adhere to over-the-counter artemisinin combination therapy for malaria? evidence from an intervention study in Uganda
Source: Malar J. 2012 Mar 23;11:83. doi: 10.1186/1475-2875-11-83 (PMC3342228; doi:10.1186/1475-2875-11-83)
Supplement: Additional file 1 — Table S1. Dosage Given by Patient Age. [file 1475-2875-11-83-S1.DOC]

| **Table A1. Dosage Given by Patient Age** | | |  |  |  |
| --- | --- | --- | --- | --- | --- |
|  | Dosage Received at Drug Shop: | | | |  |
| Patient Age | Dosage 1: Ages 3 and Under | Dosage 2: Ages 4 - 7 | Dosage 3: Ages 8 - 12 | Dosage 4: Ages 13 and Up | Dosage Given is Missing |
| 3 and Under | 91 | 4 (all age = 3) | 0 | 0 | 1 |
| 4 - 7 | 2 (both age = 4) | 47 | 2 (both age = 7) | 0 | 0 |
| 8 - 12 | 0 | 2 (age 9 and age 10) | 13 | 1 (age 12) | 0 |
| 13+ | 0 | 0 | 1 (age 13) | 93 | 1 |
